# Investigating compensatory adjustments induced by rhythmic auditory stimulation for changes in temporal gait symmetry in lower-limb prosthetic users

**DOI:** 10.1371/journal.pone.0351930

**Published:** 2026-06-26

**Authors:** Aliaa Gouda, Muhammad Zeeshan Arshad, Jan Andrysek

**Affiliations:** 1 Institute of Biomedical Engineering, University of Toronto; Holland Bloorview Kids Rehabilitation Hospital, Toronto, Canada; 2 Bloorview Research Institute, Holland Bloorview Kids Rehabilitation Hospital, Toronto, Canada; Opole University of Technology: Politechnika Opolska, POLAND

## Abstract

Gait training is an important part of rehabilitation for prosthetic users, improving mobility and long-term health outcomes. This study investigated the effects of rhythmic auditory stimulation (RAS) on gait symmetry and overall movement patterns in lower-limb prosthesis users (LLPUs). We aimed to determine how RAS, designed to enhance temporal gait symmetry, influenced overall lower-limb kinematic patterns and whether such changes were beneficial or detrimental to gait. Analysis of discrete parameters and the Gait Profile Score (GPS) indicated that while RAS improved temporal symmetry, it did not significantly alter movement patterns for both transfemoral (TFA) and transtibial amputees (TTA). Subject-specific analyses revealed varied responses, with some LLPU participants showing improved gait symmetry without significant movement changes, while others exhibited compensatory adjustments. To further explore lower-limb kinematic changes associated with temporal symmetry, a perturbation-based task involving able-bodied (AB) individuals demonstrated the presence of unique kinematic changes, including reduced ankle plantarflexion and increased hip flexion, reflecting adjustments to synchronize with the asymmetric beat. Finally, subject-specific analyses revealed user experience as a key factor influencing the LLPU response. Participants with more years of prosthesis use demonstrated greater improvements in their overall gait pattern (GPS). This study highlights that RAS can improve gait symmetry in LLPUs without inducing other significant, compensatory changes in their movement patterns, a positive finding for clinical rehabilitation. However, our results show that individual responses can vary depending on factors such as prosthesis type and user experience and these should be considered in clinical care. Future research should explore a broader range of gait parameters and long-term effects of RAS to gain a comprehensive understanding of its impact on gait and rehabilitation.

## Introduction

Gait is a complex process that demands the coordinated function of multiple systems within the body. For lower-limb prosthesis users (LLPUs), this coordination also involves a prosthetic device. New prosthesis users typically undergo training focused on regaining balance, proper limb loading, and improving movement patterns during gait. Despite initial training, abnormal gait patterns and gait deviations often persist and can worsen over time [1].

One common deviation in unilateral LLPU gait is increased reliance on the intact limb, which can generate greater propulsion and support during gait to compensate for the limitations, or perceived limitations, of the prosthetic limb [2]. Studies have reported increased ground reaction forces, joint moments, muscle activity and time spent on the intact side compared to the prosthetic side [1,2]. These compensatory mechanisms, while functional, can lead to increased metabolic energy expenditure and joint stress, resulting in long-term musculoskeletal issues such as lower back pain or joint degeneration [2–4]. Therefore, it is important to encourage LLPUs to develop the confidence and ability to more effectively utilize the prosthetic limb [5,6].

Recently, technology-based gait training, such as biofeedback and stimulation systems, has gained interest for both clinical and research applications in the rehabilitation of LLPUs [7–10]. These systems have been shown to improve LLPU gait, including increasing the time the prosthetic limb is loaded [7,9]. For example, our recent pilot study suggested that corrective feedback could improve stance time symmetry [7], and rhythmic auditory stimulation (RAS) can improve both stance time and cadence concurrently [11]. However, it remains unclear and has not been thoroughly explored in research how targeting a specific gait parameter (or multiple parameters) will impact other parameters and, consequently, overall gait patterns. For example, will an improved stance time symmetry lead to better overall gait patterns, or could it trigger undesired compensatory movements and worsen other gait parameters? [12].

Evaluating inter-dependencies between gait parameters is essential [13]. Targeting a single parameter can inadvertently affect other aspects of gait. For instance, Roerdink et al. [14] examined whether step-length asymmetry in LLPUs was a consequence of other asymmetries, including trunk progression and the forward placement of the foot relative to the trunk. Specifically, some study participants with apparently symmetrical step lengths actually had significant, opposing asymmetries in trunk progression and foot placement. The authors concluded that analyzing changes in a single gait parameter (for example step-length in their study) was insufficient and did not adequately capture important changes in overall gait symmetry.

Few studies have assessed the effects of gait training on non-targeted parameters [7,15]. For example, Darter et al. used a visual reality biofeedback system where the intervention specifically targeted frontal-plane kinematics to correct the patient’s trunk lean, pelvic drop, and hip abduction. While they found significant improvements in these targeted motions, they noted no corresponding changes in the non-targeted spatiotemporal parameters [15]. Escamilla et al. found that improvements in temporal symmetry reduced knee flexion of the prosthetic leg, when using a vibrotactile biofeedback gait training system [7]. However, that kinematic change was ultimately a result of reduced walking speed. This further highlights the interdependencies between parameters and underscores the need to understand the overall movement patterns (kinematics) and the cascading effects on targeting a single temporal parameter on gait.

As a key neurologic technique used in rehabilitation for a variety of clinical and non-clinical populations [11,16,17], RAS utilizes the physiological effects of auditory rhythm on the motor system to improve the control of movement, primarily gait [18]. While our previous research found that temporal symmetry of AB participants and LLPUs could be changed and improved using RAS [8], the broader effects on overall movement patterns remain unclear. Therefore, the main objective of this study was to determine what lower-body kinematic changes accompany these RAS-induced improvements in temporal symmetry. Specifically, it was unknown whether there were particular movements or compensatory adjustments that were elicited by the subjects to achieve changes in temporal symmetry, and how these changes affected overall gait patterns. This is important from a clinical perspective, as targeting and improving one gait parameter should ideally not worsen other aspects of gait. To better understand these untargeted kinematic changes, our study included both data from LLPUs and a reference able-bodied (AB) group to provide context for interpreting adaptation strategies.

To contextualize the kinematic changes observed in the LLPU group, we included a reference AB group. A direct comparison of symmetry improvement was not appropriate, as the AB group already exhibits near-symmetric gait and therefore has limited capacity for further improvement. Accordingly, the study was designed to examine two distinct motor adaptation tasks rather than a direct comparison of outcomes. The LLPU group was assigned an optimization task, aiming to correct a sub-optimal gait pattern toward symmetry, which reflects a common goal in rehabilitation. In contrast, the AB group was assigned a perturbation task, in which their stable, symmetric gait was intentionally disrupted to induce asymmetry. This perturbation-based approach is widely used in motor control research to investigate how the neuromuscular system adapts to externally imposed constraints [19–21]. In this context, LLPUs represent a constrained neuromechanical system, where movement is limited by prosthetic components and reduced joint function, whereas able-bodied individuals represent a relatively unconstrained system, with full biological joint control and adaptability. By examining these two forms of adaptation under matched magnitudes of temporal symmetry change (despite opposite directions of change), we aim to characterize how constrained and unconstrained systems reorganize movement to meet similar temporal demands. This framework allows us to interpret LLPU adaptations in the context of responses observed in an unconstrained system, thereby helping to distinguish compensatory strategies from those limited by prosthetic constraints.

The aim of this study was to explore the changes in gait patterns (kinematics) associated with RAS targeting temporal gait symmetry. The main research questions this study aimed to address are:

- Does RAS, as a means of changing temporal symmetry, result in significant gait pattern changes as captured through kinematics?
- If so, which kinematic parameters have the most significant changes and are these changes towards an improvement or worsening of kinematic gait patterns for LLPUs?
- What kinematic adaptation strategies are observed in the AB and LLPU groups in response to RAS?

### Methods

This prospective cross-sectional study compared gait patterns of LLPU and AB with and without RAS. The study examined the effect sizes of changes in individual gait kinematic parameters and the Gait Profile Score (GPS) for overall gait kinematics.

### Participants

This study included fourteen LLPU participants (6 females, 8 males; 32.1 ± 15.4 years; height 171 ± 9.6 cm; weight 70.9 ± 16.5 kg, 6 transfemoral and 8 transtibial; years since amputation median 12 (IQR 9.5, 16); years with their current prosthesis median 1.8 (IQR 1, 4.75)) as shown in Table 1. LLPU participants were included if they were fourteen years or older and community ambulators able to walk on level ground without additional ambulatory aids (except for current prosthetic devices for LLPU participants). The study included LLPUs with amputations at various levels (transfemoral, transtibial, Van Nes rotationplasty, knee disarticulation). All participants had no previously known neurological disorders. The study also included ten AB participants (7 females, 3 males; 25.3 ± 8.8 years; height 170.5 ± 7.91 cm; weight 66.7 ± 11.7 kg).

**Table 1:** Demographic and clinical characteristics of the LLPU group.

Characteristic	LLPU Group (n = 14)
Sex (Female / Male)	6 / 8
Age (years)^*^	32.1±15.4
Height (cm)^*^	171.0±9.6
Weight (kg)^*^	70.9±16.5
Years Post Amputation^†^	12.0 [9.5, 16.0]
Current Prosthesis (years)^†^	1.8 [1.0, 4.75]
Amputation Type (n)	
Transfemoral (TFA)	5
Transtibial (TTA)	4
Van Nes Rotationplasty	4
Knee Disarticulation	1
Prosthesis Type (n)	
Mechanical	8
Hydraulic	1
Mechanical–Hydraulic	2
Active^‡^	3

^*^ Reported as Mean ± Standard Deviation (SD).

^†^ Reported as Median [Interquartile Range (IQR)].

^‡^ Includes microprocessor-controlled, powered, and other advanced prosthetic systems.

Recruitment was facilitated through Holland Bloorview Kids Rehabilitation Hospital. LLPU participants were referred through the Prosthetics and Orthotics department and other external organizations (The War Amps and Amputee Coalition of Toronto). AB participants were recruited through posted recruitment bulletins. The study was approved (REB-0448, approved on 18 December 2021) by the Research Ethics Board at Holland Bloorview Kids Rehabilitation Hospital, Canada and the participants were recruited between December 20, 2021 and May 29, 2024. Informed written consent from each participant was obtained before conducting the study.

## Dataset – experimental protocol

The experimental protocol for the presented dataset is detailed in our previous studies and summarized here [8,11]. Participants were involved in a single gait training session. They were equipped with two systems: (1) a biofeedback system designed to provide RAS to elicit changes in temporal symmetry and (2) a wearable motion capture system to capture lower limb kinematics. The biofeedback system consisted of two triaxial inertial sensors (DOT v2, Movella North America Inc., Henderson, NV, USA), connected to an Android phone via Bluetooth. The wearable sensor-based motion capture system used was the Xsens MVN Awinda (Movella North America Inc., Henderson, NV, USA) that included seven triaxial inertial sensors. A pair of headphones was used to deliver the RAS. The RAS was designed to provide varying levels of temporal symmetry and therefore alter the stance-time symmetry ratio (STSR). The stance time for each limb was calculated as the duration between its heel-strike and its subsequent toe-off [11]. The STSR was then calculated as the ratio of both sides (prosthetic-intact or right-left), as per Equation (1), where TO represents toe-off, HS represents heel-strike events for the respective limb and i denotes the current gait cycle index. The goal of the RAS was intentionally different for the two groups. For the LLPU group (including transfemoral (TFA) and transtibial (TTA) amputees), that typically exhibits a baseline asymmetry with an STSR less than 100%, the cues were designed to improve the baseline asymmetry by directing the participants to spend more time on their prosthetic side during its stance phase as compared to the intact side, moving their STSR closer to 100%. For the AB group, whose STSR is typically near 100%, the only possible way to alter their STSR was to induce a temporal asymmetry. This was done by directing them to spend more time on their left side compared to the right side during stance phase (target STSR < 100%).


$$\begin{array}{c} STSR_{AB}=\frac{(TO_{i}-HS_{i-1}{)}_{right}}{(TO_{i}-HS_{i-1}{)}_{left}}\;\text{or}\;STSR_{LLPU}=\frac{(TO_{i}-HS_{i-1}{)}_{prosthetic}}{(TO_{i}-HS_{i-1}{)}_{intact}}, \end{array}$$ (1)

Following setup and calibration, participants were instructed to complete 2 laps at their self-selected walking speed to measure their baseline stance-time symmetry and cadence. A lap was a 15-m straight line with two 1-m radius turns. Next, the participants were coached on how to interpret the RAS. They were instructed to associate their heel-strikes with the metronome they were listening to. An acclimation period of 6–15 laps was provided until the participant confirmed they were comfortable with the task. The initial target STSR for RAS in each trial was set to the participant’s baseline STSR (for AB this was 100%) and it increased by 3–4% only when the participant’s real-time STSR remained within a defined range (target ± error) for at least 80% of 10 consecutive steps [8]. A single trial included half a lap without RAS then RAS turned on at the subsequent heel-strike of the right or prosthetic side. After 14 laps, the RAS turned off and participants continued walking for the remainder of the half lap. Additionally, to minimize the carry-over effect from the previous trial, participants were asked to walk for one to two minutes without listening to any metronomes. At the end of the session, participants were instructed to complete one lap without metronomes at their self-selected speed, considered the post-training retention trial. Data from these washout periods and the final retention trial were collected for a separate analysis of learning effects and are not included in the present cross-sectional study. In addition to the kinematic data, LLPU participants also completed the ambulation scale of the Prosthetic Evaluation Questionnaire (PEQ) at baseline, a self-reported assessment of functional outcomes and satisfaction with their prosthetic device, which provides a score out of 100.

### Data analysis

Xsens data were exported using Xsens MVN Analyze software (Xsens North America Inc., CA, USA). All the joint angles were extracted from the Xsens system and segmented into individual gait cycles (heel strike to heel strike) labelled as preRAS (immediately before RAS) or RAS. The data were then analyzed using Python and the SciPy library [22].

The dataset was split into three groups: AB, TFA, and TTA. Recognizing that the primary difference between the TFA and TTA groups is the presence of a prosthetic knee joint, we anticipated potential differences in their responses to the RAS. A critical step was to ensure a valid contextual reference for the AB group. Since the goal was to examine the kinematic strategies used to achieve a temporal change, it was essential to ensure the magnitude of this temporal change was equivalent between the LLPU and AB groups. This step ensured that both groups were exposed to a comparable magnitude of temporal perturbation. However, given the opposing direction of change, this matching does not imply equivalence of the underlying adaptation processes. Therefore, a matched AB dataset was used by selecting gait cycles that exhibited an average STSR change (5.8 ± 3.7%) that was comparable to the average change observed across the LLPU groups (5.8 ± 2.3%). Data from all participants were parsed into individual gait cycles (heel strike to heel strike).

### Overall gait changes – gait profile score

The Gait Profile Scores (GPSs) were used to assess the overall gait kinematic changes for each condition (preRAS and RAS) and each group (AB, TFA, and TTA). The GPS is a metric used to quantify the overall deviation of an individual’s gait from typical gait patterns [23]. It is derived from the Gait Variable Scores (GVSs), which measure deviations in key kinematic variables such as joint angles of the hip, knee, and ankle across multiple planes of motion. Averaging GVSs over several gait cycles provides the mean GVS for each variable. The GPS is calculated as the root mean square (RMS) of all the mean GVSs, providing a single value that reflects overall gait abnormality with lower GPS values being indicative of better gait. The GPS values for typical AB gait lie in the range of 5–6° [24], whereas for LLPUs it is higher at 9.2–10.7° [25,26]. The preRAS and RAS GPS values were calculated for both sides (right/left, prosthetic/intact) and individual sides. The baseline trials for the AB group were used as the reference dataset for the GPS calculations.

To compare the GPS between preRAS and RAS conditions, and across the AB and LLPU groups, an independent t-test was used, and Cohen’s d was used for the effect sizes, whereby d > 0.2, 0.5, 0.8, and 1.3 are interpreted as small, medium, large and very large effect sizes [27]. Furthermore, Carse et al.’s study with 60 unilateral transfemoral/knee disarticulation amputees indicated that a 1.7° change in GPS values corresponds to a change of one level in the Medicare Functional Classification Level (MFCL) [28]. The MFCL, a five-level scale (K0-K4) is commonly used for classifying functional mobility of individuals with lower limb loss [29]. Hence, 1.7° was used as a threshold to assess whether the changes in GPS would be significant enough to result in a change in MFCL.

### Discrete kinematic parameters

The joint angles (in degrees) in the sagittal plane for the lower-limb joints (ankle, knee, and hip) were extracted at specific instances during each gait cycle. This included maximum ankle plantarflexion and dorsiflexion, as well as knee and hip flexion and extension. Additionally, the symmetry for each parameter was calculated according to Equation (2) where X represents the value of a given discrete kinematic parameter (e.g., maximum ankle plantarflexion), and the subscripts right, left, prosthetic, and intact denote the corresponding limb.


$$\begin{array}{c} Symm_{AB}=\frac{X_{right}}{X_{left}}\;\text{or}\;Symm_{LLPU}=\frac{X_{prosthetic}}{X_{intact}}, \end{array}$$ (2)

Significant changes for each discrete kinematic parameter between preRAS and RAS were determined based on p<0.05 (repeated measures ANOVA) and concurrently a large effect size (partial eta squared ($\eta^{2}$)) as per [30], where $\eta^{2}>$ 0.01, 0.06, 0.14 were interpreted as small, medium, and large.

### Feature analysis

Gait data analysis presents significant challenges due to high dimensionality, temporal dependencies, variability, non-linear relationships, and inter-correlations [31]. Traditional statistical methods may overlook critical parameters or detect irrelevant changes [32,33]. In contrast, machine learning (ML) approaches can provide complementary insights into gait pattern classification by addressing these challenges. Explainable ML, has emerged to counter the “black box” nature of some ML models, providing insights into how specific data patterns influence model predictions [33–35]. For instance, tree-based models like decision trees and random forests (RF) inherently offer explainability through their architecture, utilizing metrics like Gini impurity and information gain to rank the influence of individual gait parameters [34]. Studies have demonstrated the efficacy of these methods in clinical settings, identifying significant gait parameters that traditional analysis might miss [32,34].

Hence, feature analysis was used to classify the kinematic parameters with the most observed changes. Two classifiers were selected to train the models: (1) Random Forest (RF) and (2) Support Vector Machine (SVM) for a binary classification task. The goal of this task was to classify individual gait cycles as belonging to either the preRAS or RAS condition, using the discrete kinematic parameters as input features. SVM are suitable since kinematic data often exhibit non-linear relationships. Both RF and SVM are valuable in providing insights into which features, such as kinematic parameters, are most influential in the model’s predictions, providing a complementary perspective on factors contributing to changes in gait patterns between different conditions. To determine the best hyperparameters, a 5-fold cross-validation grid search was implemented. The dataset was divided into five subsets with models trained on four subsets and validated on the remaining one. This process was repeated five times with different subsets. Grid search explores a predefined set of hyperparameter values to find the optimal combination that maximizes model performance. The performance of the model was evaluated on the basis of accuracy, precision, recall, and F1 score, as defined by Equations (3) below where TP (True Positive) is the number of RAS cycles correctly classified as RAS, TN (True Negative) is the number of preRAS cycles correctly classified as preRAS, FP (False Positive) is the number of preRAS cycles incorrectly classified as RAS, and FN (False Negative) is the number of RAS cycles incorrectly classified as preRAS. The developed models were run 10 times to find the best results and the hyperparameters presented in Table 2 were selected.


$$\begin{array}{cc} \text{Accuracy} & =\frac{TP+TN}{\text{Total Predictions}}\\ \text{Precision} & =\frac{TP}{TP+FP}\\ \text{Recall} & =\frac{TP}{TP+FN}\\ F1 & =2\times \frac{\text{Precision}\times \text{Recall}}{\text{Precision}+\text{Recall}} \end{array}$$ (3)

**Table 2:** Optimized hyperparameters for best model performance.

Model	Hyper-parameter Space	Best (AB)	Best (LLPU)
RF	max_depth: [15,25,35]	max_depth = 25	max_depth = 35
	n_estimators: [50, 100, 150]	n_estimators = 150	n_estimators = 100
	min_samples_split: [3,5,8]	min_samples_split = 5	min_samples_split = 5
	max_leaf_nodes: [50, 100, 150]	max_leaf_nodes = 100	max_leaf_nodes = 150
	max_features: [auto, sqrt, log2]	max_features = sqrt	max_features = sqrt
SVM	kernel: [linear, poly, rbf, sigmoid]	kernel = rbf	kernel = linear
	C: [100, 10, 1.0, 0.1, 0.001]	C = 1.0	C = 100
	gamma: [scale, auto]	gamma: auto	gamma = scale

Finally, the importance values of the characteristics were extracted for each model (using the Gini impurity for the RF and the hyperplane coefficient for SVM) [34,36] to identify which kinematic gait parameters change the most significantly between the preRAS and RAS conditions.

## Results

### Gait profile score

The analysis of Gait Profile Score (GPS) across the LLPU groups indicated that RAS did not significantly change movement patterns while improving symmetry (Table 3). In the AB group, significant changes in GPS were observed with comparable STSR changes (p<0.02, d = 0.968). The AB group exhibited a larger magnitude of GPS change than the TTA (p = 0.044, d = 1.066) and TFA (p = 0.05, d = 1.138) groups. AB GPS score increased by 1.46 ± 1.42°, while TFA and TTA changed by only −0.03 ± 0.82° and 0.24 ± 0.44°, respectively.

**Table 3:** Gait Profile Scores in degrees (°) for each condition and group. Pink and blue shading indicate significant differences (p<0.05) and large effect sizes (Cohen’s d > 0.8), respectively. Positive and negative changes in GPS (difference between RAS and preRAS) indicate gait moving further away or closer to typical AB gait, respectively.

Group	Side	preRAS	RAS	Pairwise Comparison (preRAS vs RAS)	
		Mean ± Std		p-value	Effect Size (Cohen’s d)
**AB**	Right	4.31 ± 1.29	6.0 ± 2.41	0.039	0.82
	Left	4.51 ± 1.1	5.73 ± 1.48	0.012	1.076
	Both	4.45 ± 1.07	5.9 ± 1.88	0.02	0.968
**TFA**	Prosthetic	12.97 ± 4.56	12.83 ± 3.56	0.837	−0.088
	Intact	8.63 ± 2.19	8.77 ± 2.46	0.681	0.178
	Both	11.06 ± 3.44	11.03 ± 2.91	0.938	−0.033
**TTA**	Prosthetic	10.46 ± 1.47	10.41 ± 1.53	0.773	−0.106
	Intact	10.48 ± 3.15	10.97 ± 3.53	0.076	0.735
	Both	10.54 ± 2.11	10.78 ± 2.34	0.196	0.505
**Group**		**Difference (RAS-preRAS)**		**AB vs LLPU (Difference)**	
		**Mean ± Std**		**p-value**	**Effect Size (Cohen’s d)**
**AB**		1.46 ± 1.42		—	—
**TFA**		−0.03 ± 0.82		0.05	1.138
**TTA**		0.24 ± 0.44		0.044	1.066

Based on the subject-specific GPS analysis, there were notable differences in the magnitude and nature of changes between the groups. For the LLPU participants (Table 4), none had a change greater than 1.7° for the overall GPS score, which is considered a clinically significant threshold as outlined by Carse et al. [28]. However, the GPS per side score along with the GVS joint data varied among these participants. Specifically, 5 of the 14 LLPU participants had a clinically significant change (greater than 1.7° in magnitude) for at least one joint-level GVS score. As shown in Table 4, these changes varied in direction; for example, participant P13 experienced a large improvement (a negative change of −4.89°) in prosthetic knee GVS, while participant P11 experienced a large worsening (a positive change of 3.70°) in prosthetic hip GVS. Overall, for the TFA and TTA groups, there were varying responses at the joint level. The AB group exhibited larger overall changes. As expected, RAS inducing temporal asymmetry for this group resulted in GPS and GVS further away from typical AB gait for most of the participants (Table 5). Significantly, 3 of the 10 AB participants exceeded the 1.7° clinically significant threshold for their overall GPS score.

**Table 4:** GPS and GVS in degrees (°) for each LLPU participant, limb side and joint angle. Positive and negative values indicate GPS score moved away from or towards typical AB gait, respectively. Green indicates gait is improving (light green: >-1.7°, green: <-1.7°) and red indicates it is worsening (light red: <1.7°, red: >1.7°).

Group	ID	Prosthesis Type	Gait Profile Scores			Gait Variability Score					
			Overall	Overall Pros.	Overall Intact	Pros. Ankle	Intact Ankle	Pros. Knee	Intact Knee	Pros. Hip	Intact Hip
**TTA**	P01	Mechanical	0.04	−0.81	0.88	0.61	0.06	−1.82	1.43	−0.32	0.76
	P02	Mechanical	0.63	0.36	0.85	0.09	1.29	0.60	−0.10	0.36	1.53
	P03	Hydraulic	−0.55	−0.63	−0.46	0.09	−0.55	−1.35	−1.08	−0.63	0.47
	P04	Mechanical	−0.05	0.12	−0.24	0.49	0.12	0.48	−0.13	−0.46	−0.69
	P06	Mechanical	0.40	0.24	0.53	0.17	−0.34	0.18	0.79	0.33	0.97
	P09	Mechanical	−0.01	0.02	−0.08	1.19	0.35	−1.33	0.01	1.22	−0.56
	P10	Mechanical	0.53	−0.19	1.17	0.01	1.20	−1.16	1.12	1.26	1.22
	P11	Mechanical	0.92	0.52	1.26	−0.01	0.07	−2.13	2.14	3.70	1.02
**TFA**	P05	Active	0.93	0.59	1.30	0.56	0.56	0.73	1.17	0.94	1.94
	P07	Active	−0.06	0.19	−0.49	0.44	−0.56	−0.83	−0.54	1.04	−0.34
	P08	Active	0.97	1.81	−0.35	0.98	−0.71	2.45	−0.63	1.93	0.76
	P12	Mechanical	−0.17	−0.11	−0.26	−0.82	−1.56	0.28	−1.32	−0.32	1.56
	P13	Hydraulic	−1.42	−2.86	1.02	−0.27	0.35	−4.89	0.43	−1.87	3.28
	P14	Hydraulic	−0.41	−0.43	−0.38	−0.02	0.17	−0.70	−0.63	−0.39	−0.42

**Table 5:** GPS and GVS in degrees (°) for each AB participant, limb side and joint angle. Positive and negative values indicate GPS score moved away from or towards typical AB gait, respectively. Green indicates gait is improving (light green: >-1.7°, green: <-1.7°) and red indicates it is worsening (light red: <1.7°, red: >1.7°).

ID	Gait Profile Scores (°)			Gait Variability Score (°)					
	Overall	Overall Right	Overall Left	Right Ankle	Left Ankle	Right Knee	Left Knee	Right Hip	Left Hip
P01	0.63	0.32	0.98	0.77	1.04	0.05	0.55	0.39	0.85
P02	2.91	0.87	1.91	4.41	4.99	3.16	0.78	3.02	2.79
P03	1.02	−0.1	1.30	3.27	1.09	−0.56	0.83	0.95	1.08
P04	4.63	7.60	3.47	7.40	1.47	2.61	5.75	6.17	3.17
P05	1.24	0.63	0.16	1.94	0.72	1.71	3.00	1.30	1.17
P06	0.22	−0.16	1.03	−1.31	1.77	−0.60	0.65	−0.64	1.11
P07	1.19	1.40	1.02	1.63	0.24	2.02	1.69	0.27	1.36
P08	1.76	1.02	1.54	4.80	0.42	3.16	−0.74	2.68	0.51
P09	−0.36	0.02	−1.56	0.56	0.77	−0.91	−0.40	−0.06	−0.56
P10	1.09	0.10	−0.17	1.31	0.14	3.38	2.59	1.34	0.87

**Fig 1:**
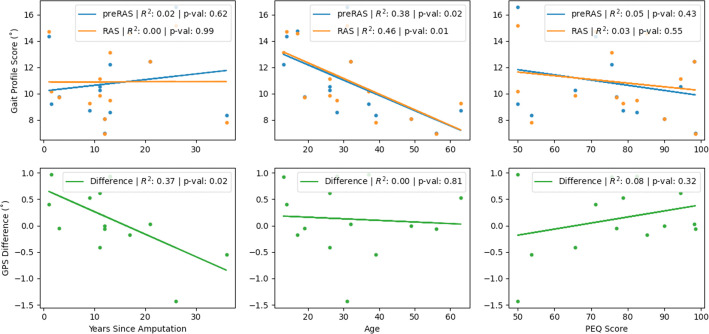
Gait Profile Scores (GPS) and GPS Difference relative to years since amputation and age for LLPU group based on linear regression analysis; For top row, Y-axis represent absolute GPS where lower values= moving closer to typical AB gait and higher values = moving away from typical AB gait. For bottom row, Y-axis represents the GPS Difference (RAS - preRAS) where positive values = moving away from typical AB gait, negative values = moving closer to typical AB gait.

**Fig 2:**
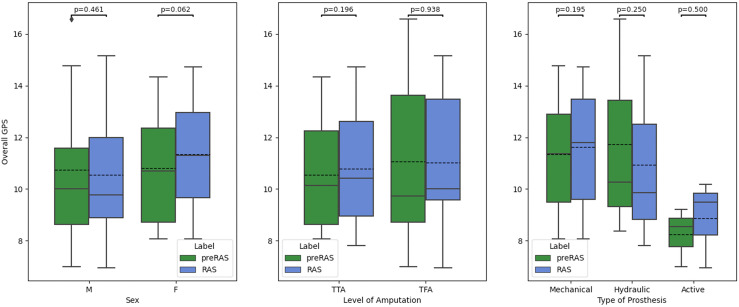
Gait Profile Scores relative sex, level of amputation and type of prosthesis.

For the LLPU group, Fig 1 shows the relationships between the Gait Profile Score (GPS) and three variables including years since amputation, age, and PEQ Score, for both preRAS and RAS conditions. For years since amputation, both preRAS and RAS show weak and non-significant relationships, but the GPS difference demonstrates a significant decrease with more years since amputation (r² = 0.37, p = 0.02). For age, there are weak but statistically significant relationships for both preRAS (r² = 0.38, p = 0.02) and RAS (r² = 0.46, p = 0.01), suggesting a slight improvement in GPS with increasing age. However, the GPS difference shows no significant relationship with age (r² < 0.001, p = 0.81). The PEQ Score was not significantly correlated with the GPS in either the preRAS (p = 0.43) or RAS (p = 0.55) conditions, nor with the GPS difference (p = 0.32). The boxplots in Fig 2 illustrate the overall GPS for different groups categorized by sex, level of amputation, and type of prosthesis, preRAS and RAS conditions. Comparing preRAS and RAS conditions, none of the groups exhibited significant differences.

### Discrete kinematic gait parameters

For the discrete kinematic parameters, the TFA and TTA groups had several parameters that differed significantly, however, the effect size was not large ($\eta^{2}<0.14$), as per the findings presented in Table 6. Whereas the AB group exhibited significantly different parameters with a larger overall effect size. The statistical analysis results for the complete datasets (including preRAS and RAS conditions) can be found in Table S1 Table, S2 Table, S3.

**Table 6:** Difference in discrete kinematic parameters between preRAS and RAS conditions for each group, side (right/prosthetic, left/intact) and symmetry. Blue shade indicates parameters with significant difference between preRAS and RAS conditions with large effect size ($\eta^{2}>0.14$). Statistical analysis (including p-value and effect size is included in the appendices). R – right, L – left, SR – symmetry ratio, P – prosthetic, I – intact.

Joint	Parameter	AB (Mean ± Std)			TTA (Mean ± Std)			TFA (Mean ± Std)		
		R	L	SR	P	I	SR	P	I	SR
**Ankle**	Dorsiflexion	2.4 ± 0.52	1.33 ± 0.87	0.28 ± 0.12	0.51 ± 0.3	1.07 ± 0.24	0.07 ± 0.04	0.56 ± 0.21	0.71 ± 0.33	0.08 ± 0.05
	Plantarflexion	6.28 ± 1.74	2.22 ± 1.2	0.2 ± 0.10	0.56 ± 0.53	3.49 ± 0.68	0.07 ± 0.06	0.58 ± 0.13	1.72 ± 1.31	0.02 ± 0.01
	Sagittal ROM	4.06 ± 1.29	2.16 ± 0.99	0.05 ± 0.03	0.86 ± 0.24	4.52 ± 0.98	0.06 ± 0.02	0.38 ± 0.36	1.49 ± 1.75	0.02 ± 0.03
**Knee**	Initial Flexion	1.47 ± 0.33	1 ± 0.51	0.07 ± 0.03	2.04 ± 2.4	1.81 ± 1.31	0.06 ± 0.1	3.77 ± 0.68	2.42 ± 0.77	0.1 ± 0.07
	Terminal Stance Extension	1.39 ± 0.3	1.89 ± 0.62	0.32 ± 0.15	0.36 ± 0.37	1.23 ± 0.61	0.18 ± 0.11	0.16 ± 0.31	0.63 ± 0.48	0.07 ± 0.13
	Max Flexion	0.98 ± 0.82	1.13 ± 0.55	0.03 ± 0.01	1.21 ± 0.27	1.26 ± 0.43	0.01 ± 0.01	4.06 ± 0.8	0.57 ± 0.48	0.06 ± 0.01
	Sagittal ROM	2.58 ± 1.15	1.07 ± 0.67	0.03 ± 0.02	2.41 ± 0.34	2.4 ± 0.56	0.02 ± 0.01	4.23 ± 1.1	1.33 ± 0.47	0.06 ± 0.02
**Hip**	Extension	1.88 ± 0.43	2.6 ± 0.66	0.2 ± 0.1	1.2 ± 0.25	1.74 ± 0.32	0.33 ± 0.16	1.29 ± 0.94	1.82 ± 0.86	0.11 ± 0.09
	Flexion	1.05 ± 0.38	2.4 ± 0.37	0.07 ± 0.02	0.98 ± 0.23	1.51 ± 0.28	0.04 ± 0.01	1.58 ± 0.4	2.19 ± 0.55	0.04 ± 0.01
	Sagittal ROM	2.35 ± 0.46	1.2 ± 0.65	0.04 ± 0.02	1.87 ± 0.47	2.91 ± 0.58	0.06 ± 0.02	2.1 ± 0.87	1.5 ± 0.64	0.06 ± 0.01

### Machine learning – feature analysis

**Fig 3:**
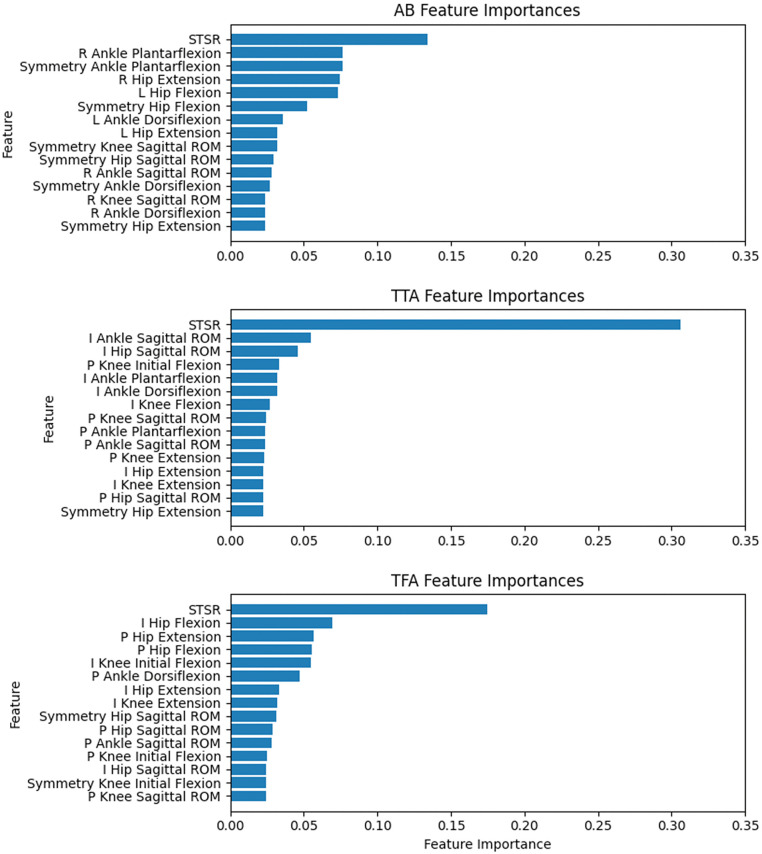
Top 15 features ranked based on extracted feature importance values of RF models for AB, TTA and TFA groups.

Both RF and SVM models performed well (>90%) when classifying the different conditions (preRAS and RAS), with RF performing the best (Table 7). Consequently, feature importance values for the RF models were extracted for each group, as illustrated in Fig 3. STSR is the most significant feature for all groups, with its highest importance in the TTA group, followed by the TFA and AB groups. This is expected, since STSR was the directly targeted parameter. For the AB group, while STSR is still relevant, other parameters such as ankle plantarflexion and hip extension also play substantial roles.

**Table 7:** ML models performance metrics (accuracy, precision, recall, and F1 scores).

Group	Model	Accuracy (%)	Precision	Recall	F1 Score
**AB**	RF	95.2 ± 1.5	95.8 ± 1.2	94.7 ± 1.2	95.8 ± 1.3
	SVM	90.7 ± 1.5	90.8 ± 1.5	90.7 ± 1.5	90.8 ± 1.4
**TFA**	RF	94.1 ± 1.2	94.3 ± 1.2	94.1 ± 1.2	94.2 ± 1.2
	SVM	92.1 ± 2.2	92.3 ± 2.1	92.1 ± 2.2	92.1 ± 2.2
**TTA**	RF	96.8 ± 0.7	96.8 ± 0.7	96.8 ± 0.7	96.8 ± 0.7
	SVM	95.0 ± 1.8	95.2 ± 1.7	95.0 ± 1.8	95.0 ± 1.8

## Discussion

This study aimed to determine whether compensatory adjustments, captured through kinematics, were generated due to elicited changes in temporal symmetry using rhythmic auditory stimulation (RAS).

A key consideration in interpreting the findings is the fundamentally different objectives assigned to the AB and LLPU groups. While the LLPU group was guided toward improving an existing asymmetry (an optimization task), the AB group was intentionally perturbed away from their baseline symmetry (a perturbation task). As such, the direction and purpose of adaptation should be considered in the interpretation of the results. Specifically, the AB group was not intended to serve as a normative benchmark, but rather as a mechanistic reference to illustrate how an unconstrained neuromechanical system adapts to imposed temporal changes. Accordingly, comparisons in this study are intended to contrast adaptation strategies under differing task constraints, rather than to directly equate outcomes between groups.

For the LLPU group (both TTA and TFA), analysis of GPS, GVS and discrete gait parameters showed that improvements in stance time symmetry with RAS did not significantly change overall movement patterns; rather STSR gains were achieved through small, localized adjustments without clear compensatory changes, a finding supported by the feature-based analysis (Fig 3), which identified STSR as the most important feature for distinguishing preRAS and RAS conditions. In contrasts, the AB group demonstrated significant changes during the perturbation task, including a 1.46° change in GPS [28] and significant alterations in several discrete parameters at the hip and ankle joints. The fact that STSR improved without corresponding changes in global kinematics, particularly in the LLPU cohort, is encouraging as it suggests temporal symmetry can be enhanced without inducing abnormal gait patterns. However, it also indicates that the intervention did not improve pre-existing gait abnormalities, as indicated by the unchanged GPS. The highly targeted nature of the auditory cue may allow participants to achieve the temporal goal without needing to fundamentally reorganize their overall kinematic strategy. This likely reflects the highly targeted nature of auditory cues or cuing, which allows participants to meet the temporal objective without fundamentally reorganizing their kinematics strategy, and aligns with prior research [7,14,15] showing that improvements in one aspect of gait do not necessarily translate to broader kinematic changes and may influence other domains in non-uniform ways.

The machine learning analysis further extends these findings by providing a multivariate, system-level perspective on gait adaptation, complementing traditional statistical approaches by capturing interactions that may not be evident when parameters are examined independently. The high classification accuracy (90%, Table 7) demonstrates that preRAS and RAS conditions are clearly separable based on the selected feature set. More importantly, the feature importance distributions (Fig 3) highlight distinct differences in adaptation strategies across groups: the AB group showed a broader contribution from secondary kinematic features, such as ankle plantarflexion and hip extension, suggesting a more distributed, whole-limb response to the perturbation, whereas, both TTA and TFA groups exhibited a steep decline in feature importance beyond STSR, with minimal contribution from other kinematic variables. This pattern suggests that while the AB system adapts through coordinated changes across multiple joints, adaptation in LLPUs remains more localized and constrained, likely reflecting prosthetic and biomechanical limitations.

It is also important to consider the unique and varied responses of individual LLPUs. GPS analysis (Tables 4 and 5) indicated that while some AB participants surpassed the 1.7° clinically significant threshold [28], none of the LLPU participants did. However, the GPS (per side scores) and GVS joint data showed varied responses among LLPU participants. Notably, 5 out of 14 LLPU participants experienced changes greater than ±1.7° in at least one joint GVS, mainly at the prosthetic hip and knee. Therefore, while the overall analysis showed few kinematic differences, these results show that individual changes could still be clinically significant. This heterogeneity is further highlighted by our finding from Fig 1 that more experienced LLPUs (those with more years since amputation) demonstrated a greater improvement in their overall GPS in response to RAS. Given that LLPU gait is primarily influenced by the level of limb loss and the type of prostheses, making them a highly heterogenous group [37], the application of gait training interventions such as RAS must take these individual factors into account in clinical practice. Clinicians should be encouraged to consider and assess overall gait changes, recognizing that a user’s experience with their prosthesis may influence their ability to make gait improvements using RAS (Fig 1).

Understanding the mechanisms underlying changes in STSR is essential for interpreting how adaptation strategies differ under varying task constraints; although no consistent trends were observed across the LLPU groups, the AB group demonstrated the most substantial changes in ankle and hip kinematics, as reflected by the large effect sizes (Table 6) and the feature importance values illustrated in Fig 3. Specifically the reduction in the right limb ankle plantarflexion during RAS likely reflects a strategy to slow the right limb during swing, thereby increasing stance phase duration on the left side; this reduced ankle plantarflexion is consistent with diminished push-off requirement at lower swing velocities [38]. Additionally, the increase in hip flexion on the left side likely occurred to prolong the stance phase duration, to achieve temporal asymmetry. These adjustments allowed participants to synchronize with the asymmetrical beat provided by RAS. Similar adjustments were not observed in the LLPU group due possibly to the restricted ankle range of motion and movement, as all LLPU participants used passive fixed-ankle prostheses, limiting control over joint response and adjustments. This suggests that LLPUs may achieve temporal changes through more subtle or distributed adjustments that do not register as large-effect changes in any single discrete parameter.

The finding that more experienced LLPU participants demonstrated greater improvements warrants further discussion, particularly in the context of the AB group’s response. Though there is limited research in this area, our results align with Seth et al., who reported that better functional mobility can be linked to time since amputation [39]. A plausible explanation for this is enhanced motor adaptability. Specifically, it is plausible that those who have used prostheses longer have developed a more flexible motor schema, allowing them to more efficiently integrate external cues like RAS to optimize their gait pattern. Furthermore, their greater experience may also reduce the cognitive load of walking, freeing up resources to focus on the auditory task. While the magnitude of this improvement in experienced LLPUs is modest compared to the absolute magnitude of change seen in the AB group, its positive direction (an improvement vs. a worsening) is significant. In contrast, the AB group showed the greatest negative change (a GPS increase), likely because they have no experience with gait compensation. Their task was not to optimize a sub-optimal pattern, but to disrupt a highly stable, efficient one. This adaptability in LLPUs, however, may be influenced by the type of amputation. For instance, the TFA group, even with a similar capacity for compensation as the TTA group, might be more limited by the control and function of the prosthetic knee joint.

One limitation of this study is the lack of additional gait parameters data, such as upper-body movements or kinetic measures. Without these additional parameters, the analysis may overlook critical aspects of gait dynamics that could influence the findings. Future studies should aim to include a broader range of gait parameters. Additionally, investigating whether these increased movements lead to higher metabolic cost, muscle activity, or other physiological impacts, can provide a deeper insight into the functional implications of gait variations. Furthermore, future research should also investigate these changes in individuals who have only recently acquired their prosthesis. These individuals would likely be less familiar with compensatory mechanisms, providing a clearer view of the initial adaptations and challenges faced during the early stages of prosthetic use. This study assessed only the short-term compensatory changes, and it remains unclear whether kinematics would change further with longer term use of the RAS system. Finally, differences in task objectives and baseline symmetry between AB and LLPU groups limit direct comparison of outcomes, and findings should be interpreted within the context of differing adaptation goals.

## Conclusions

This study demonstrated that rhythmic auditory stimulation (RAS) effectively improved temporal symmetry in gait for both TFA and TTA with only minimal compensatory changes in their overall movement patterns, captured through kinematics. Minimal kinematic changes were observed in the LLPU groups, whereas more pronounced adjustments were observed in the AB reference group. The variations in individual responses in LLPU group, particularly influenced by prosthetic type and user experience, highlight the complexity of gait adjustments and the nuanced impact of RAS on different amputee populations. Notably, improvements in temporal symmetry with minimal compensatory changes suggest that RAS can enhance gait symmetry without causing abnormal gait patterns, a positive outcome for rehabilitation efforts. However, the study’s limitations, such as the exclusion of upper-body and kinetic data, suggest that future research should include a broader range of gait parameters and explore long-term effects to provide a more comprehensive understanding of gait dynamics and prosthetic use.

## Supporting information

S1 TableIndividual kinematic gait parameters for each condition and side for the AB group, including statistical analyses (p-values and partial eta squared/effect size).R – right side, L – left side, SR – symmetry ratio. Orange and green highlights represent decreases and increases compared to baseline, respectively (significantly different parameters only). Pink and blue shading represent parameters that were statistically significantly different from baseline and exhibited a large effect size (($\eta^{2}>0.14$)).(PDF)

S2 TableIndividual kinematic gait parameters for each condition and side for the TFA group, including statistical analyses (p-values and partial eta squared/effect size).P – prosthetic side, I – intact side, SR – symmetry ratio. Orange and green highlights represent decreases and increases compared to baseline, respectively (significantly different parameters only). Pink and blue shading represent parameters that were statistically significantly different from baseline and exhibited a large effect size (($\eta^{2}>0.14$)).(PDF)

S3 TableIndividual kinematic gait parameters for each condition and side for the TTA group, including statistical analyses (p-values and partial eta squared/effect size).P – prosthetic side, I – intact side, SR – symmetry ratio. Orange and green highlights represent decreases and increases compared to baseline, respectively (significantly different parameters only). Pink and blue shading represent parameters that were statistically significantly different from baseline and exhibited a large effect size (($\eta^{2}>0.14$)).(PDF)
