# Supplementary material for: Investigating compensatory adjustments induced by rhythmic auditory stimulation for changes in temporal gait symmetry in lower-limb prosthetic users
Source: PLoS One. 2026 Jun 26;21(6):e0351930. doi: 10.1371/journal.pone.0351930 (PMC13308856; doi:10.1371/journal.pone.0351930)
Supplement: S1 Table — R – right side, L – left side, SR – symmetry ratio. Orange and green highlights represent decreases and increases compared to baseline, respectively (significantly different parameters only). Pink and blue shading represent parameters that were statistically significantly different from baseline and exhibited a large effect size ((η2>0.14)). (PDF) [file pone.0351930.s001.pdf]

Table S1. Individual kinematic gait parameters for each condition and side for the AB group, including statistical analysis (p-values and partial eta squared/effect size). R – right side, L – left side, SR – symmetry ratio. Orange and green highlights represented decrease and increase compared to baseline (only significantly different parameters). Pink and blue shading represent statistically significantly different compared to baseline and large effect size ($\eta^2 > 0.14$).

Gait Parameter		Able-Bodied Participants (n=10)											
		Mean (± Standard Deviation Values)						Pairwise Comparison (p-Values)			Pairwise Comparison (Partial Effect Size η^2)		
		preRAS (R)	preRAS (L)	preRAS (SR)	RAS (R)	RAS (L)	RAS (SR)	preRAS - RAS (R)	preRAS - RAS (L)	preRAS - RAS (SR)	preRAS - RAS (R)	preRAS - RAS (L)	preRAS - RAS (SR)
Stance-Time Symmetry Ratio (%)		—	—	98.892.93	—	—	93.332.92	—	—	0.004	—	—	0.504
Ankle	Dorsiflexion	15.663.82	14.523.26	1.10.23	17.863.6	13.834.31	1.360.37	0.054	0.289	0.027	0.11	0.012	0.255
	Plantarflexion	-19.89.35	-23.518.81	0.860.29	-13.597.45	-22.348.38	0.660.35	0.013	0.307	0.003	0.152	0.006	0.153
	Sagittal ROM	35.477.74	38.037.2	0.940.12	31.536.04	36.486.73	0.880.16	0.02	0.149	0.011	0.087	0.015	0.056
Knee	Initial Flexion	33.222.95	33.13.47	1.010.09	33.923.55	32.463.36	1.050.13	0.332	0.495	0.306	0.018	0.005	0.055
	Terminal Stance Extension	1.293.69	2.092.99	1.240.7	2.193.15	2.623.24	1.150.71	0.087	0.523	0.794	0.027	0.014	0.003
	Max Flexion	67.333.87	66.934.49	1.010.03	66.663.95	67.245.31	0.990.05	0.07	0.537	0.192	0.01	0.002	0.048
	Sagittal ROM	71.375.36	70.534.6	1.010.04	69.034.83	69.795.17	0.990.06	0.002	0.15	0.039	0.077	0.006	0.081
Hip	Extension	-9.662.95	-9.762.3	10.25	-8.113.8	-7.373.61	1.060.44	0.088	0.059	0.419	0.053	0.203	0.018
	Flexion	29.382.7	29.092.3	1.010.06	29.43.32	31.343.32	0.940.1	0.782	0.031	0.022	0.001	0.168	0.204
	Sagittal ROM	39.143.32	38.912.66	1.010.05	37.554.19	38.762.48	0.970.1	0.077	0.49	0.132	0.058	0.008	0.078
