# Supplementary material for: Investigating compensatory adjustments induced by rhythmic auditory stimulation for changes in temporal gait symmetry in lower-limb prosthetic users
Source: PLoS One. 2026 Jun 26;21(6):e0351930. doi: 10.1371/journal.pone.0351930 (PMC13308856; doi:10.1371/journal.pone.0351930)
Supplement: S2 Table — P – prosthetic side, I – intact side, SR – symmetry ratio. Orange and green highlights represent decreases and increases compared to baseline, respectively (significantly different parameters only). Pink and blue shading represent parameters that were statistically significantly different from baseline and exhibited a large effect size ((η2>0.14)). (PDF) [file pone.0351930.s002.pdf]

Table S2. Individual kinematic gait parameters for each condition and side for the TFA group, including statistical analysis (p-values and partial eta squared/effect size). P – prosthetic side, I – intact side, SR – symmetry ratio. Orange and green highlights represented decrease and increase compared to baseline (only significantly different parameters). Pink and blue shading represent statistically significantly different compared to baseline and large effect size ($\eta^2 > 0.14$).

Gait Parameter		Transfemoral Amputees (TFA) Participants (n=6)												
		Mean (± Standard Deviation Values)							Pairwise Comparison (p-Values)			Pairwise Comparison (Partial Effect Size η^2)		
		preRAS (AB)	preRAS (P)	preRAS (I)	preRAS (SR)	RAS (P)	RAS (I)	RAS (SR)	preRAS - RAS (P)	preRAS - RAS (I)	preRAS - RAS (SR)	preRAS - RAS (P)	preRAS - RAS (I)	preRAS - RAS (SR)
Stance-Time Symmetry Ratio (%)		—	—	—	89.839.31	—	—	96.48.59	—	—	< 0.001	—	—	0.161
Ankle	Dorsiflexion	14.313.48	15.031.95	10.683.2	1.450.38	15.282.32	9.983.21	1.550.36	0.676	0.029	0.005	0.001	0.02	0.029
	Plantarflexion	-22.17.87	3.364.68	-24.325.22	0.190.13	3.885.36	-25.046.17	0.20.13	0.496	0.77	0.87	0.001	0.001	0.001
	Sagittal ROM	36.417.11	16.921.72	35.195.35	0.490.05	16.951.9	35.416.6	0.490.08	0.877	0.656	0.483	0.001	0.001	0.016
Knee	Initial Flexion	32.913.6	15.68.25	28.774.55	0.560.3	14.886.21	27.084.48	0.580.29	0.68	0.001	0.664	0.002	0.044	0.002
	Terminal Stance Extension	3.282.12	0.181.93	5.093.92	0.560.6	0.291.76	4.513.73	0.590.62	0.501	0.346	0.995	0.004	0.004	0.001
	Max Flexion	66.484.04	51.7320.24	62.283.38	0.830.32	53.617.86	62.933.46	0.850.28	0.511	0.047	0.587	0.002	0.009	0.001
	Sagittal ROM	71.184.45	52.0120.41	61.564.28	0.830.31	53.7118	62.113.77	0.850.26	0.605	0.801	0.575	0.001	0.001	0.002
Hip	Extension	-10.343.04	-14.525.67	-4.862.86	1.840.36	-13.435.94	-4.153.03	1.820.38	0.114	0.333	0.674	0.014	0.034	0.005
	Flexion	28.942.94	27.8910.06	325.97	0.920.42	29.6510.04	33.925.87	0.930.4	0.045	0.022	0.832	0.005	0.028	0.001
	Sagittal ROM	39.333.12	42.7813.65	37.145.15	1.20.46	43.8813.32	38.525.23	1.190.44	0.605	0.026	0.345	0.001	0.02	0.001
