# Supplementary material for: Investigating compensatory adjustments induced by rhythmic auditory stimulation for changes in temporal gait symmetry in lower-limb prosthetic users
Source: PLoS One. 2026 Jun 26;21(6):e0351930. doi: 10.1371/journal.pone.0351930 (PMC13308856; doi:10.1371/journal.pone.0351930)
Supplement: S3 Table — P – prosthetic side, I – intact side, SR – symmetry ratio. Orange and green highlights represent decreases and increases compared to baseline, respectively (significantly different parameters only). Pink and blue shading represent parameters that were statistically significantly different from baseline and exhibited a large effect size ((η2>0.14)). (PDF) [file pone.0351930.s003.pdf]

Table S3. Individual kinematic gait parameters for each condition and side for the TTA group, including statistical analysis (p-values and partial eta squared/effect size). P – prosthetic side, I – intact side, SR – symmetry ratio. Orange and green highlights represented decrease and increase compared to baseline (only significantly different parameters). Pink and blue shading represent statistically significantly different compared to baseline and large effect size ($\eta^2 > 0.14$).

Gait Parameter		Transtibial Amputees (TTA) Participants (n=8)												
		Mean (± Standard Deviation Values)							Pairwise Comparison (p-Values)			Pairwise Comparison (Partial Effect Size η^2)		
		preRAS (AB)	preRAS (P)	preRAS (I)	preRAS (SR)	RAS (P)	RAS (I)	RAS (SR)	RAS (P)	RAS (I)	RAS (SR)	preRAS - RAS (P)	preRAS - RAS (I)	preRAS - RAS (SR)
Stance-Time Symmetry Ratio (%)		—	—	—	91.897.86	—	—	96.836.57	—	—	< 0.001	—	—	0.143
Ankle	Dorsiflexion	14.313.48	14.822.97	15.635.35	1.040.36	14.863.14	15.895.91	1.060.41	0.954	0.791	0.719	< 0.001	< 0.001	< 0.001
	Plantarflexion	-22.17.87	-0.772.98	-23.756.65	0.110.09	-0.353.08	-21.048.35	0.170.21	0.025	0.148	0.179	0.007	0.045	0.084
	Sagittal ROM	36.417.11	20.133.98	40.998.22	0.520.18	20.14.4	37.179.45	0.580.21	0.785	0.042	0.077	< 0.001	0.059	0.035
Knee	Initial Flexion	32.913.6	23.757	32.114.13	0.760.3	23.058.63	31.155.69	0.770.37	0.338	0.242	0.671	0.003	0.027	< 0.001
	Terminal Stance Extension	3.282.12	-2.455.15	2.822.59	0.940.75	-2.155.16	1.932.71	0.920.75	0.067	0.117	0.535	0.001	0.067	0.003
	Max Flexion	66.484.04	58.135.71	64.64.57	0.910.11	56.115.15	65.866.85	0.860.1	0.137	0.351	0.041	0.039	0.008	0.044
	Sagittal ROM	71.184.45	63.778.04	68.883.98	0.930.14	60.327.39	68.797.53	0.890.15	0.021	0.973	0.095	0.044	0.003	0.019
Hip	Extension	-10.343.04	-11.957.59	-8.478.21	1.280.48	-11.067.56	-7.248.46	1.260.57	0.238	0.187	0.917	0.003	0.005	0.001
	Flexion	28.942.94	29.718.66	32.757.5	0.90.09	30.489.05	33.257.52	0.910.1	0.236	0.428	0.61	0.001	0.001	0.002
	Sagittal ROM	39.333.12	41.818.59	41.375.19	1.010.16	41.719.76	40.87.19	1.020.15	0.964	0.59	0.501	0.001	0.002	0.002
